# The Negative Association Between Positive Psychological Wellbeing and Loss Aversion

**DOI:** 10.3389/fpsyg.2021.641340

**Published:** 2021-03-18

**Authors:** Ibuki Koan, Takumi Nakagawa, Chong Chen, Toshio Matsubara, Huijie Lei, Kosuke Hagiwara, Masako Hirotsu, Hirotaka Yamagata, Shin Nakagawa

**Affiliations:** Division of Neuropsychiatry, Department of Neuroscience, Yamaguchi University Graduate School of Medicine, Ube, Japan

**Keywords:** decision making, gamble, loss aversion, psychological wellbeing, personal growth, positive psychology

## Abstract

When making decisions, people tend to overweigh the impact of losses compared to gains, a phenomenon known as loss aversion (LA). A moderate amount of LA may be adaptive as it is necessary for protecting oneself from danger. However, excessive LA may leave people few opportunities and ultimately lead to suboptimal outcomes. Despite frequent reports of elevated LA in specific populations such as patients with depression, little is known about what psychological characteristics are associated with the tendency of LA. Based on the neurobiological studies of LA, we hypothesized that positive psychological wellbeing may be negatively associated with people's tendency of LA. In the present study, we set out to test this hypothesis in a sample of young adults. We evaluated LA using a decision-making task in which subjects were asked to decide whether to accept or reject a series of coin-toss gambles. Our results revealed that individuals with more advanced personal growth as assessed by the Ryff's Psychological Well-being Inventory showed reduced LA. To our knowledge, this is the first report demonstrating an association between positive psychological wellbeing and LA. These findings suggest that personal growth might be employed as interventional targets for correcting excessive LA in vulnerable populations.

## Introduction

When making decisions, people tend to overweigh the impact of losses compared to gains (Kahneman and Tversky, 1979). For instance, when presented with gambles with equal chances of winning and losing, in order to accept a gamble, people demand almost twice the amount of potential gains compared to losses (Tom et al., 2007). This phenomenon, known as loss aversion (LA), has been an extensively studied decision bias (Barberis, 2013, but see Yechiam and Hochman, 2013; Gal and Rucker, 2018; Yechiam, 2019 for critiques of the concept).

Generally speaking, a moderate amount of LA may be adaptive and preferred since it is necessary for protecting oneself from danger (Li et al., 2012). In contrast, in certain situations, excessive LA may lead to negative, undesirable consequences. Excessive LA may prevent people from exploring the environment and leave people few potentially profitable opportunities. Previous research has indicated elevated LA in individuals with childhood trauma (Huh et al., 2016), depression (Chandrasekhar Pammi et al., 2015; Huh et al., 2016; Engelmann et al., 2017) (in particular depressed patients with a history of suicide attempt, see Baek et al., 2017), and obsessive-compulsive disorder (Sip et al., 2018). Healthy subjects also show increased LA in response to negative stimuli such as unpleasant odors (Stancak et al., 2015) or fearful faces (Schulreich et al., 2016).

Despite these findings, little is known about what psychological characteristics predict people's tendency of LA. This knowledge would be rather valuable, given that the identified psychological characteristics may be employed as interventional targets for correcting excessive LA in vulnerable populations. It may also help us achieve a better understanding of the psychological mechanism of LA.

Based on the neurobiological studies of LA, we hypothesized that positive psychological wellbeing may be negatively associated with people's tendency of LA. Specifically, the amygdala and the striatum have been suggested as the neural substrate of LA. For instance, the possibility of financial losses activates the amygdala and striatum (Krawczyk and D'Esposito, 2013) and increases the functional connectivity between these two brain areas (Charpentier et al., 2015). Furthermore, neural LA (i.e., the slope of the decrease in activation for increasing losses being greater than the slope of the increase in activation for increasing gains) has been detected in the ventral striatum (Tom et al., 2007). In contrast, bilateral amygdala lesions eliminate behavioral LA (De Martino et al., 2010).

Importantly, a previous study reported that when confronted with potentially aversive information, individuals with more positive psychological wellbeing, as evaluated by the Ryff's Psychological Well-being Inventory (Ryff, 1989), show reduced activation in the amygdala (Van Reekum et al., 2007). Meanwhile, they also show increased activation in the ventral anterior cingulate cortex (ACC). The ventral ACC and its closely related ventromedial prefrontal cortex are believed to exert cognitive control and emotion regulation through modulating the activation of the amygdala and ventral striatum (Ochsner et al., 2012; Jhang et al., 2018). Therefore, it may be speculated that people with more positive psychological wellbeing are better able to inhibit their activation of the amygdala and striatum in response to potential losses, therefore demonstrating reduced LA. This speculation is consistent with a recent report that individuals with greater psychological wellbeing use more rational, adaptive decision-making strategies (Páez-Gallego et al., 2020). In the present study, we set out to investigate the association between LA and psychological wellbeing in a sample of healthy young adults.

## Materials and Methods

### Participants

This research was part of an ongoing larger study, the aim of which was to predict future mental health status in healthy young adults using high-level cognitive functions. The study was carried out in accordance with the latest version of the Declaration of Helsinki and approved by the Institutional Review Board of Yamaguchi University Hospital. Subjects were recruited via posters placed on campus and department homepage. Data of 33 subjects collected at the baseline of the study for the first author's thesis research were used for the analysis here. Seven subjects had to be excluded due to too many gamble acceptances (95.30 ± 2.40%, *n* = 6) or zero gamble acceptance (*n* = 1) in the LA task, which hinders reliable estimation of the LA coefficient (see below). The data of the remaining 26 subjects were used for the present analysis. Notably, including six of the excluded subjects for whom we were able to obtain an estimated LA coefficient (reliability aside), the negative association between personal growth and LA coefficient identified in this study remained unchanged (*n* = 32; for Pearson correlation: *r* = −0.319, *p* = 0.038, one-tailed test; for linear regression with all six domains of psychological wellbeing as the independent variables and LA coefficient as the dependent variable, only personal growth was significant and the unstandardized coefficient *B* = −0.049, *p* = 0.034, one-sided test); for details of this data, please see the Supplementary Figure 1.

All subjects agreed to participate in this study and provided written informed consent after receiving a detailed explanation of the study. Subjects were well-informed that they were free to decline to participate and withdraw from the study at any point after agreeing to participate if they wished. They were also well-informed that their names would be withheld when data is reported. The inclusion criteria were being 20–39 years old at the time of the visit and the exclusion criteria were (1) having any self-reported mental health disorders, (2) receiving medical examinations due to suspicion of any mental health disorders, (3) being suspected of mental health disorders by the research staff and subsequently diagnosed as having a mental health disorder by the Mini-International Neuropsychiatric Interview conducted by a psychiatrist, or (4) being unable to perform the laboratory tests and answer the questionnaires for this study due to severe physical conditions or other reasons. No participant was excluded owing to meeting any of the exclusion criteria.

### Measures and Loss Aversion Task

Subjects first answered questions about their demographic characteristics, including gender, age, occupation, education level, and socioeconomic status such as their personal income (for students, this is their monthly allowance plus their part-time earnings if any), whether they had made a student loan, their parents' education levels and family income.

**Figure 1:**
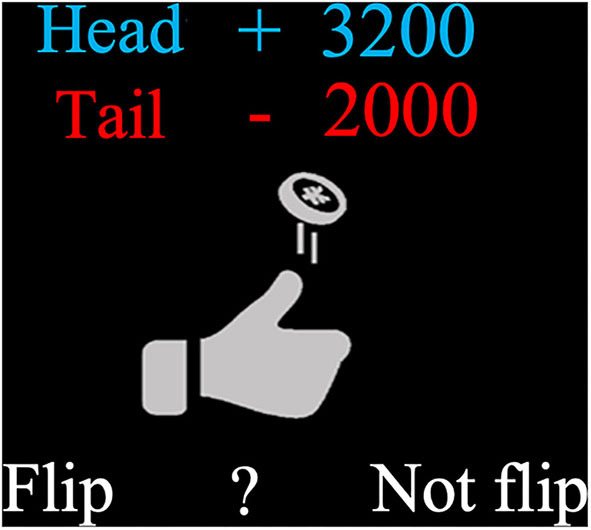
Illustration of a typical trial. On each trial, subjects had to decide whether to accept or reject a coin-toss gamble that offered equal (50%) chances of gaining or losing different amounts of money.

We used an established experimental paradigm (Tom et al., 2007; De Martino et al., 2010) to evaluate LA. The task was programmed using MATLAB R2018b (MathWorks) and Psychtoolbox 3 (http://psychtoolbox.org/). In this task, subjects were asked to decide whether to accept or reject a series of 64 coin-toss gambles that offered equal (50%) chances of gaining or losing different amounts of money (Figure 1). If the head of the coin faced up, the subject would win a variable amount of money (shown in blue); if the tail faced up, the subject would lose a variable amount of money (shown in red). Gambles included 64 different pairs of combinations, which were created by randomly sampling without replacement from a matrix with possible gambles consisting of eight levels of gains (from 2000 to 4800 incremented in steps of 400, all JPY) and eight levels of losses (from 1000 to 2400 incremented in steps of 200). The purpose of employing this asymmetric gain/loss matrix was to be more sensitive around the typical indifference points (i.e., losses are weighted about twice as strongly as gains, Tom et al., 2007; He et al., 2010; Gelskov et al., 2016) to efficiently capture subjects' LA tendency. Before the task, subjects practiced four trials to get familiar with the task and response keys.

Subjects were told that they would receive about 3,000 JPY (roughly $30 USD) for participating in this research and they were asked to imagine that the payment would be adjusted based on their choices (they were fully aware that this would not be actually implemented): at the end of the experiment one trial would be randomly selected and subjects' actual decision in that trial would be used to adjust their payment. Specifically, if they accepted the gamble in that trial, the gamble would be executed. If the head faced up, they would receive an additional gain equal to the amount shown together with the head in that trial. However, if the tail faced up, the amount shown along with the tail in that trial would be subtracted from their original payment of 3,000 JPY. If they rejected the gamble in that trial, their payment 3,000 JPY would remain unchanged.

In this study, each subject received a fixed payment of ~3,000 JPY for participating in this study. We did not actually implement the performance-adjusted payment based on a randomly selected trial but instead asked subjects to imagine vividly that kind of payment being executed. It was because our final objective was to develop useful tools for predicting mental health problems in a public health setting: it is impossible to pay people a certain amount of money based on their choices in any public health predictive tools. Meanwhile, previous research has shown that people's decision-making with hypothetic rewards highly resembles that with real rewards (Kühberger et al., 2002; Madden et al., 2003) (See Hertwig and Ortmann, 2001 for an extensive discussion of this issue).

We calculated the LA coefficient (λ) for each subject using the following equation:

(1) $$\begin{array}{r} \lambda =-\frac{\beta_{loss}}{\beta_{win}} \end{array}$$

here, β_loss_ and β_win_ are the unstandardized regression coefficients for the loss and gain variables, respectively, estimated from logistic regression for each subject to predict their choices of acceptance or rejection (De Martino et al., 2010; Charpentier et al., 2015). λ = 1 indicates loss neutral, λ > 1 loss averse, and λ < 1 gain seeking. The logistic regression was estimated using the *fitglm* function in MATLAB R2018b. Bigger λ reflects higher LA. Furthermore, to investigate whether higher LA led to poor performance (i.e., reward outcomes) in the present study, following the instruction explained to subjects, we randomly selected one trial for each subject and calculated their adjusted payment (see above). We repeated this process 100 times and used the mean adjusted payment for each subject as their performance reward.

We used the Japanese version (Kitamura et al., 2004) of the extensively employed 84-item Ryff's Psychological Well-being Inventory (Ryff, 1989). This scale assesses six domains of psychological wellbeing: autonomy, environmental mastery, personal growth, positive relations with others, purpose in life, and self-acceptance. Each domain was measured with 14 items of statements rated on a 6-point Likert scale ranging from 1 (“strongly disagree”) to 6 (“strongly agree”). Autonomy evaluates the degree of self-determination and independence. Environmental mastery measures the sense of mastery and competence in managing one's environment. Personal growth measures the feeling of continued psychological development as well as the tendency to pursue such development. The domain of positive relations with others evaluates to what extent an individual has warm, satisfying, and trusting relationships with others. Purpose in life evaluates to what extent one has goals in life and a sense of direction. Self-acceptance evaluates to what extent one possesses a positive attitude toward the self.

### Personality, Mood, and Emotion Regulation Measures

We also considered the potential involvement of personality, mood, and emotion regulation in the association between psychological wellbeing and LA. We used the Behavioral Inhibition System and Behavioral Activation System scales (BIS/BAS, Carver and White, 1994; Takahashi et al., 2007) to assess four biological personality traits of BIS, drive, fun seeking, and reward responsiveness. The BIS measures individuals' response to aversive stimuli such as anxiety, fear, and worry. We used the Beck Depression Inventory-II (BDI-II, Beck et al., 1996; Kojima and Furukawa, 2003) to assess depressive symptoms that occurred within the past 2 weeks and the Y-1 subscale of the State–Trait Anxiety Inventory (STAI, Spielberger et al., 1983; Hidano et al., 2000) to assess state anxiety. Lastly, we used the Emotion Regulation Questionnaire (ERQ, Gross and John, 2003; Yoshizu et al., 2013) to assess individual differences in their use of two emotion regulation strategies, reappraisal and suppression.

### Data Analysis

The statistical analysis was conducted with IBM SPSS Statistics 26.0 and MATLAB R2018b. As already described, we fitted a logistic regression to estimate each subject's LA coefficient λ. We examined the relation of λ with psychological wellbeing and other measures using Pearson correlation, Student's *t*-test, and linear regression. For data analysis, income was coded as 1 for ~50,000JPY (upper bound not included), 2 for 50,000~100,000JPY, 3 for 100,000~150,000JPY, and 4 for 150,000~200,000JPY. Parents' education level was coded as 1 for elementary school level, 2 for junior high school level, 3 for senior high school level, 4 for vocational school level, 5 for undergraduate level, 6 for master's level, and 7 for doctorate level. Family income (annual) was coded as 1 for ~2,000,000 JPY (upper bound not included), 2 for 2,000,000~4,000,000 JPY, 3 for 4,000,000~6,000,000 JPY, 4 for 6,000,000~8,000,000 JPY, 5 for 8,000,000~10,000,000 JPY, 6 for 10,000,000~15,000,000 JPY, 7 for 15,000,000~20,000,000 JPY, and 8 for 20,000,000~ JPY. *P* < 0.05 was considered statistically significant. Since we predicted a negative association between psychological wellbeing and LA, one-tailed tests were conducted. Statistical power analysis was conducted for linear regression models and single regression coefficients using G^*^Power version 3.1.9.7 (Faul et al., 2009).

## Results

The sample consisted of 26 subjects, with a mean age of 23.56 years (SD 3.06 years). The mean score of each measure is shown in Table 1.

**Table 1:** The score of each measure and their association with λ.

**Measure**	**Mean ± SD**	**Association^a^ with λ**
**Demographics**		
Gender (male/female)	7/19	*t*_(24)_ = 0.558, *p* = 0.582
Age (years)	23.56 ± 3.06	*r* = −0.047, *p* = 0.820
Personal income	2.00 ± 1.02	*r* = 0.195, *p* = 0.340
Student loan (no/yes)	19/7	*t*_(24)_ = −0.571, *p* = 0.573
Father's education	5.04 ± 1.10	*r* = −0.369, *p* = 0.070
Mother's education	4.31 ± 0.88	*r* = −0.378, *p* = 0.057
Family income	4.54 ± 2.49	*r* = 0.016, *p* = 0.939
**Psychological wellbeing**		
Autonomy	49.31 ± 9.12	*r* = −0.099, *p* = 0.315
Environmental mastery	54.62 ± 8.10	***r*** **= −0.464**, ***p*** **= 0.008**
Personal growth	58.35 ± 9.15	***r*** **= −0.592**, ***p*** **= 0.001**
Positive relations with others	60.35 ± 8.15	*r* = −0.079, *p* = 0.350
Purpose in life	57.19 ± 9.41	***r*** **= −0.357**, ***p*** **= 0.037**
Self-acceptance	51.04 ± 11.52	*r* = −0.284, *p* = 0.080
**Personality (BIS/BAS)**		
BAS drive	11.77 ± 2.10	*r* = −0.328, *p* = 0.102
BAS fun seeking	12.92 ± 6.25	*r* = −0.080, *p* = 0.699
BAS reward responsiveness	16.69 ± 2.28	***r*** **= −0.395**, ***p*** **= 0.046**
BIS	20.96 ± 5.18	*r* = −0.012, *p* = 0.956
**Mood**		
Depression (BDI)	7.88 ± 5.38	*r* = 0.122, *p* = 0.553
State Anxiety (STAI)	37.19 ± 7.90	*r* = 0.142, *p* = 0.488
**Emotion regulation (ERQ)**		
Reappraisal	28.88 ± 5.23	*r* = −0.175, *p* = 0.392
Suppression	13.04 ± 4.46	*r* = −0.204, *p* = 0.316

a *Student's t-test for gender and student loan, Pearson correlation for all other measures; One-tailed test for psychological wellbeing, two-tailed for all other measures. n = 25 for income (excluding one none-student subject) and father's education (one missing data), n = 26 for all other measures. p <0.05 is indicated in bold*.

**Figure 2:**
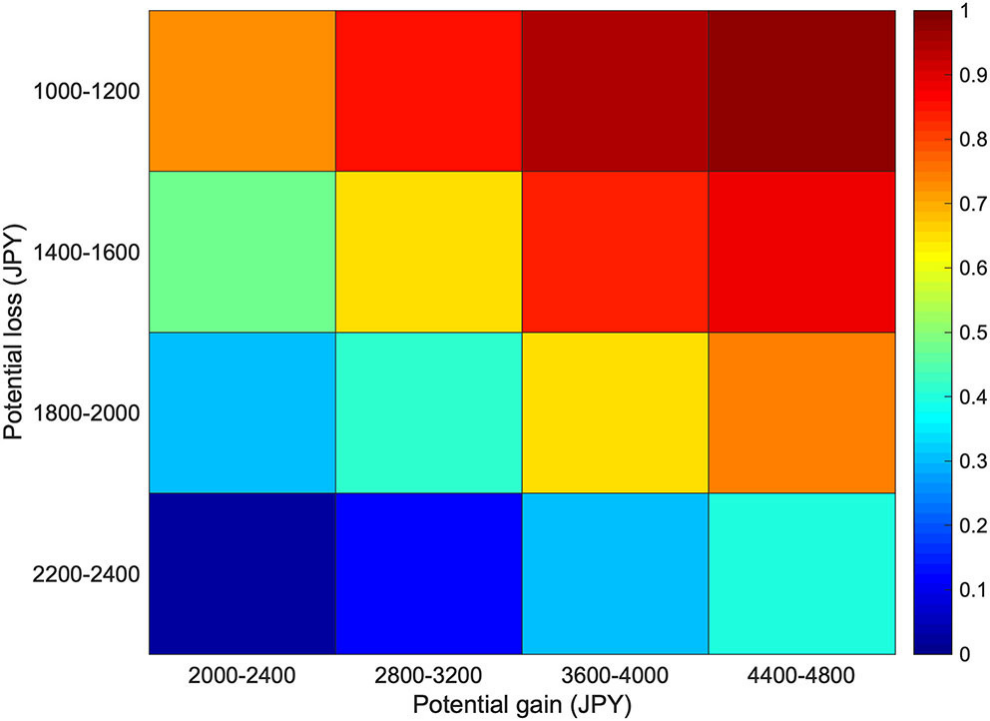
Probability of gamble acceptance. Color-coded heatmaps depicting the probability of gamble acceptance at each level of gain/loss within that cell averaged across all subjects (*n* = 26). One cell consists of four potential gain/loss combinations. Red indicates high willingness to accept the gamble, and blue indicates low willingness to accept the gamble.

On average, subjects accepted 57.86 ± 23.05% of the gambles. We collapsed the 8 by 8 gain/loss combinations into a 4 by 4 matrix and calculated the probability of gamble acceptance at each cell (which thus consists of four gain/loss combinations) for each subject. The color-coded heatmaps depicting the probability of gamble acceptance averaged across all subjects are shown in Figure 2. As can be seen, on average, subjects were indifferent to gambles (indicated in green colored cells) in which the potential gain was somewhat less than twice the amount of the potential loss. We fitted a logistic regression model for each subject to estimate their loss aversion coefficient λ. On average, the subjects had a mean λ of 1.93 (SD = 0.68, range 1.17–3.87), comparable to previous reports (Tom et al., 2007; He et al., 2010; Gelskov et al., 2016).

As we were interested in what domain(s) of psychological wellbeing might be associated with LA, we next examined the correlation between the six domains of psychological wellbeing and λ (Table 1). Specifically, we found three domains that were negatively correlated with λ, environmental mastery (*r* = −0.464, *p* = 0.008, one-tailed test), personal growth (*r* = −0.592, *p* = 0.001, one-tailed test), and purpose in life (*r* = −0.357, *p* = 0.037, one-tailed test), with the first two domains remaining significant after Bonferroni correction (0.05/6).

To confirm the association, we included all six domains in a linear regression to predict λ (**model 1**). The model was significant [*F*_(6, 19)_ = 3.118, *p* = 0.027, *R*^2^ = 0.496] and only personal growth (*B* = −0.073, *p* = 0.0045, one-tailed test) and autonomy (*B* = 0.031, *p* = 0.045, one-tailed test) were significant in predicting λ. The statistical power for this linear regression (model 1) was 0.920. The statistical power for the regression coefficients of personal growth and autonomy were 0.950 and 0.646, respectively.

**Figure 3:**
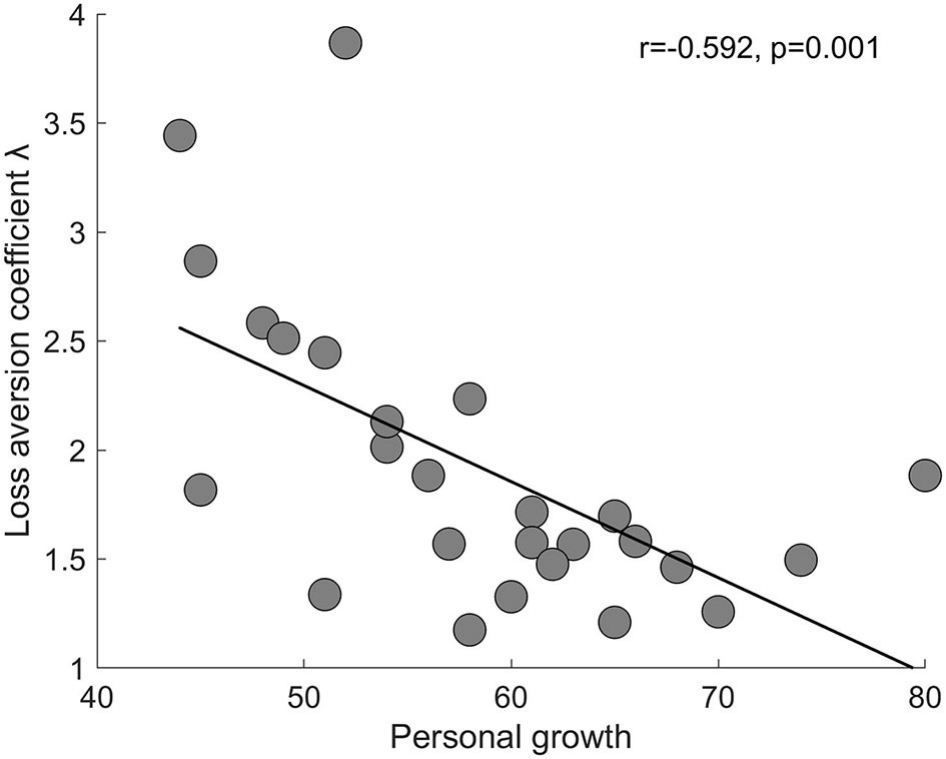
Scatter plot (with a regression line) of the association between personal growth and LA coefficient λ. Each circle represents a subject. *n* = 26.

To rule out the confounding of demographic factors that may affect decision-making and LA, we conducted a second linear regression analysis using personal growth and autonomy to predict λ, adding gender, age, personal income, student loan, father's education, mother's education, and family income as covariates (**model 2**). The model remained significant [F_(9, 15)_ = 2.584, *p* = 0.050, *R*^2^ = 0.608] and only personal growth was significant in predicting λ (*B* = −0.062, *p* = 0.0015, one-tailed test). The statistical power for this linear regression (model 2) was 0.961. Thus, across model 1 and 2, a negative association between personal growth and λ was consistently identified with a high statistical power. A scatter plot of this association is shown in Figure 3.

To investigate the potential involvement of personality, mood, and emotion regulation in the association between personal growth and LA, we conducted three additional linear regression models using personal growth to predict λ, with **model 3** adding personality measures (i.e., drive, fun seeking, reward responsiveness, and BIS) as covariates, **model 4** adding mood measures (i.e., depression and anxiety) as covariates, and **model 5** adding emotion regulation measures (i.e., reappraisal and suppression) as covariates. All three models remained significant [F_(5, 20)_ = 3.137, *p* = 0.030, *R*^2^ = 0.440 for model 3; *F*_(3, 22)_ = 4.068, *p* = 0.019, *R*^2^ = 0.357 for model 4; F_(3, 22)_ = 4.089, *p* = 0.019, *R*^2^ = 0.358 for model 5] and in all three models, only personal growth significantly predicted λ (*B* = −0.041, *p* = 0.005 for model 3; *B* = −0.045, *p* = 0.0015 for model 4; *B* = −0.042, *p* = 0.003 for model 5; all one-tailed tests). The statistical power for model 3–5 were 0.881, 0.842, and 0.844, respectively.

**Figure 4:**
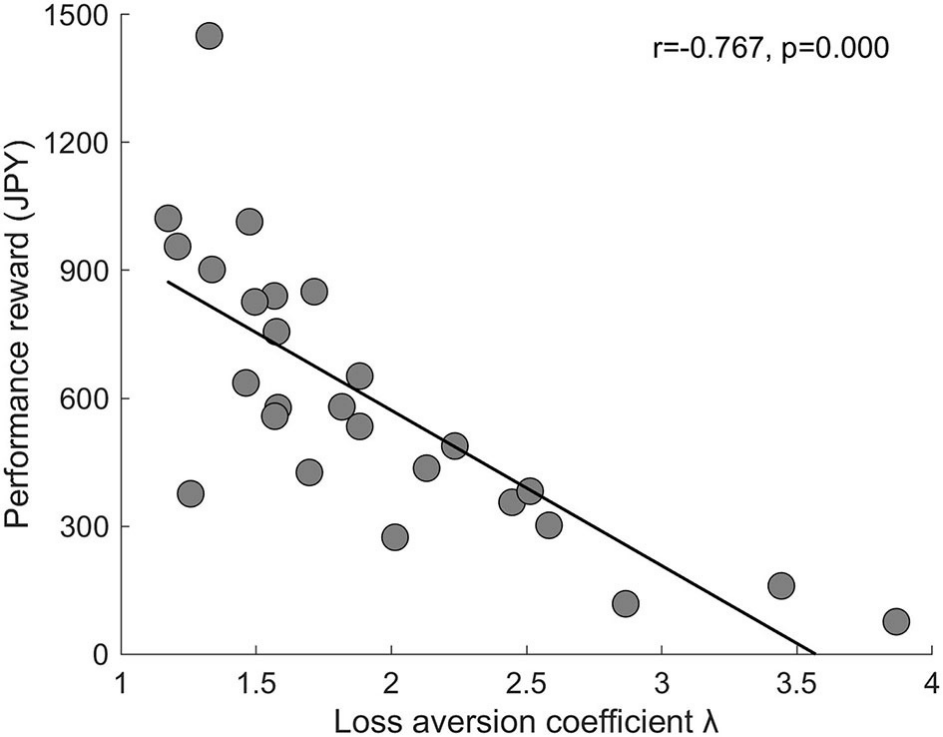
Scatter plot (with a regression line) of the association between LA coefficient λ and performance reward (JPY). Each circle represents a subject. *n* = 26.

Finally, to investigate whether excessive LA is associated with poor reward outcomes, we conducted a correlation analysis between LA coefficient λ and performance reward. The correlation was significant (*r* = −0.767, *p* = 0.000) such that greater LA was associated with fewer performance reward (Figure 4). We further confirmed that among the six dimensions of psychological wellbeing, only personal growth was significantly correlated with performance reward (*r* = 0.392, *p* = 0.024, one-tailed test) and this correlation disappeared after partialling out λ (*r* = −0.120, *P* = 0.284, one-tailed test). This indicates that the positive association between personal growth and performance reward was driven by LA.

## Discussion

In line with our hypothesis, in a sample of healthy young adults, we found one specific domain of psychological wellbeing, personal growth, is negatively associated with LA. More advanced personal growth is associated with reduced LA. This association stayed stable even after controlling demographic factors and considering the influence of personality, mood, and emotion regulation measures. Furthermore, greater LA is associated with fewer performance reward in our task. Individuals with higher personal growth have reduced LA, and this gains them more performance reward. To our knowledge, this is the first report demonstrating an association between positive psychological wellbeing measures and LA.

Here, personal growth measures an individual's sense of continued psychological development (or improvement) as well as the tendency to pursue such development, for instance, through having new experiences that challenge oneself or broaden one's horizons (Ryff, 1989). It is possible that this kind of seeing growth in oneself and being open to new experiences restrains one's tendency to overweigh the impact of losses compared to gains. This speculation is consistent with the observation that individuals with more positive psychological wellbeing including personal growth show increased activation in the ventral ACC and reduced activation in the amygdala when confronted with potentially aversive information (Van Reekum et al., 2007). In other words, these individuals demonstrate typical brain activations that indicate reduced LA.

While we observed such an association, the precise underlying mechanism remains uninvestigated. In contrast to the original LA theory that emphasizes a decision mechanism of overweighing losses over gains, several authors have recently argued that LA is driven by attention rather than decision mechanisms (e.g., Yechiam and Hochman, 2013; Lejarraga et al., 2019). It remains for future studies to probe whether individuals with more advanced personal growth are better able to balance their attention toward potential losses and gains, or they have a more balanced decision strategy while considering losses and gains simultaneously. Still, a third possibility might be that individuals with more advanced personal growth are better able to use a secondary, positive reappraisal strategy during decision-making (Balzarotti et al., 2016). This is in line with another recent report that when explicitly instructed to use a “regulate” strategy by considering each choice in a bigger context, people show reduced LA accompanied by increased activation in the PFC and reduced activation in the amygdala (Sokol-Hessner et al., 2013).

The recent positive psychology movement (e.g., Seligman, 2012) has suggested the possibility of continued psychological development and growth. It is stimulating to see whether the improvement of such personal growth can result in reduced LA, which may be further employed as interventional targets for correcting excessive LA in vulnerable populations.

A limitation of the present study is that we used a small sample of young healthy participants and excluded seven subjects in our main analysis whose LA could not be reliably estimated. Although the statistical power of the association we identified was high, further investigation is required to confirm the association in samples with a bigger sample size and broader age range as well as clinical samples with known excessive LA. Another limitation is that we used just mixed gambles from an asymmetric gain/loss matrix and did not include gain-only gambles that allow us to disentangle the specific effect of LA from that of diminishing sensitivity to payoff magnitude (Yechiam and Hochman, 2013; Walasek and Stewart, 2015) or more general risk-aversion (e.g., De Martino et al., 2010; Sokol-Hessner et al., 2013). Future research is required to overcome this limitation and provide further mechanical accounts of the association we found and the association between self-reported happiness and risk-taking behavior in situations involving losses identified by a previous study (Yechiam et al., 2016).

Although it has been reported that gender (Schmidt and Traub, 2002) and culture (Wang et al., 2017) may affect loss aversion, we did not find such a gender difference in loss aversion in our small Japanese sample. Future well-designed studies may investigate whether such gender and cultural differences exist in loss aversion and in the association between loss aversion and personal growth.

In summary, in a sample of healthy young adults, we found that individuals with more advanced personal growth as evaluated by the Ryff's Psychological Well-being Inventory show reduced LA. To our knowledge, this is the first report demonstrating an association between positive psychological wellbeing and LA. These findings suggest that personal growth might be employed as interventional targets for correcting excessive LA in vulnerable populations.

## Data Availability Statement

The dataset that support the findings of this study are available from the corresponding author upon reasonable request.

## Ethics Statement

The studies involving human participants were reviewed and approved by Institutional Review Board of Yamaguchi University Hospital. The patients/participants provided their written informed consent to participate in this study.

## Author Contributions

CC, HL, and SN: conceptualization and methodology. IK, TN, CC, TM, KH, and MH: investigation. IK and CC: formal analysis and writing—original draft preparation. IK, TN, CC, TM, HL, KH, MH, HY, and SN: writing—review and editing. All authors read and approved the final manuscript.

## Conflict of Interest

The authors declare that the research was conducted in the absence of any commercial or financial relationships that could be construed as a potential conflict of interest.

## Supplementary Material

Supplementary Figure 1 Scatter plot (with a regression line) of the association between personal growth and LA coefficient λ without removing subjects with high gamble acceptances. Each circle represents a subject. *n* = 32. The mean of λ is 1.75 (SD 0.72) and the median 1.57 (IQR 1.22–2.10).
