# Supplementary figures and images for: The Negative Association Between Positive Psychological Wellbeing and Loss Aversion
Source: Front Psychol. 2021 Mar 18;12:641340. doi: 10.3389/fpsyg.2021.641340 (PMC8012665; doi:10.3389/fpsyg.2021.641340)

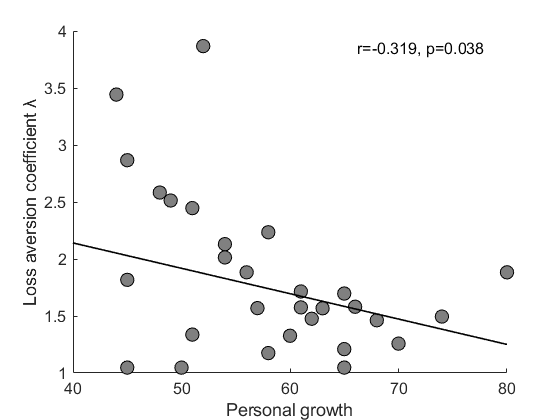

Supplement: Supplementary Figure 1 — Scatter plot (with a regression line) of the association between personal growth and LA coefficient λ without removing subjects with high gamble acceptances. Each circle represents a subject. n = 32. The mean of λ is 1.75 (SD 0.72) and the median 1.57 (IQR 1.22–2.10). [file Image_1.tif]
